# Decellularization of the mouse ovary: comparison of different scaffold generation protocols for future ovarian bioengineering

**DOI:** 10.1186/s13048-019-0531-3

**Published:** 2019-06-22

**Authors:** Ahmed Baker Alshaikh, Arvind Manikantan Padma, Matilda Dehlin, Randa Akouri, Min Jong Song, Mats Brännström, Mats Hellström

**Affiliations:** 1 Laboratory for Transplantation and Regenerative Medicine, Sahlgrenska Academy, University of Gothenburg, Göteborg, Sweden; 2 Department of Obstetrics and Gynecology, Sahlgrenska Academy, University of Gothenburg, Göteborg, Sweden; 3 Division of Gynecologic Oncology, Department of Obstetrics and Gynecology, Daejeon St. Mary’s Hospital, The Catholic University of Korea, Daejeon, South Korea; 4 Stockholm IVF-EUGIN, Stockholm, Sweden; 5 Kvinnokliniken, Blå stråket 6, SE-413 45 Göteborg, Sweden

**Keywords:** Ovary, Decellularization, Tissue engineering, Extracellular matrix, Scaffold, Biomaterial, Malignancy

## Abstract

**Background:**

In order to preserve fertility in young women with disseminated cancer, e.g. leukemia, an approach that has been suggested is to retransplant isolated small follicles within an ovarian matrix free from malignant cells and with no risk for contamination. The present study evaluates the first step to create a bioengineered ovarian construct that can act as growth-supporting tissue for isolated small follicles that are dependent on a stroma for normal follicular maturation. The present study used the intact mouse ovary to develop a mouse ovarian scaffold through various protocols of decellularization.

**Material and methods:**

Potential Immunogenic DNA and intracellular components were removed from whole mouse ovaries by agitation in a 0.5% sodium dodecyl sulfate solution (Protocol 1; P1), or in a 2% sodium deoxycholate solution (P2) or by a combination of the two (P3). The remaining decelluralized ovarian extracellular matrix structure was then assessed based on the DNA- and protein content, and was further evaluated histologically by haematoxylin and eosin-, Verhoeff’s van gieson- (for elastin), Masson’s trichrome- (for collagens) and Alcian blue (for glycosaminoglycans) staining. We also evaluated the decellularization efficiency using the mild detergent Triton-X100 (1%).

**Results:**

Sodium dodecyl sulfate efficiently removed DNA and intracellular components from the ovarian tissue but also significantly reduced the integrity of the remaining ovarian extracellular matrix. Sodium deoxycholate, a considerably milder detergent compared to sodium dodecyl sulfate, preserved the ovarian extracellular matrix better, evident by a more distinct staining for glycosaminoglycan, collagen and elastic fibres. Triton-X100 was found ineffective as a decellularization reagent for mouse ovaries in our settings.

**Conclusions:**

The sodium dodecyl sulfate generated ovarian scaffolds contained minute amounts of DNA that may be an advantage to evade a detrimental immune response following engraftment. The sodium deoxycholate generated ovarian scaffolds had higher donor DNA content, yet, retained the extracellular composition better and may therefore have improved recellularization and other downstream bioengineering applications. These two novel types of mouse ovarian scaffolds serve as promising scaffold-candidates for future ovarian bioengineering experiments.

## Introduction

Recent advancements in cancer therapy have significantly improved survival rates, but fertility dysfunction after therapy is common due to negative side effects. Concerning women and cancer therapy, even low doses of radiation significantly reduce the number of primordial follicles [1, 2]. In addition, the ovaries are particularly sensitive to alkylating cytotoxic drugs [3], and chemotherapies have been associated with vascular damage and ovarian cortical fibrosis [4, 5]. Hence, these gonadotoxic effects can lead to premature ovarian failure (POF), with accompanying early menopause and infertility. It is therefore important to consider quality-of-life after treatment, including fertility preservation [6, 7]. Current fertility preservation methods for cancer patients include embryo/oocyte vitrification, ovarian transposition and ovarian cortex transplantation [8–10]. Ovarian cortex transplantation is an effective method of fertility preservation and can potentially restore fertility in most female cancer survivors [11].

However, fertility is particularly difficult to restore in young females with hematopoietic cancer types, such as leukemia, since ovarian cortex transplantation is related to a very high risk of re-introduction of malignant cells that may be spread in the ovarian tissue, particularly in connection with the microvascularity [12, 13]. For these reasons, multiple groups investigated if isolated preantral follicles can be stimulated to growth in vitro with the aim to develop techniques to preserve fertility [14–17]. Small, preantral follicles can be isolated from ovarian cortical tissue and these follicles do not include any blood cells and are thus free from any malignant cells. Follicles may therefore be considered safe to transplant back to the patient after cancer treatment. However, the follicles cannot survive and mature without an appropriate environment that supports follicular growth. Therefore, it has been proposed to use a biomaterial as supporting material that facilitates normal follicular maturation [18–21]. Using ovarian scaffolds derived from fibrin- and/or alginate matrices, or three-dimensional (3D) printed structures of cross-linked gelatine, several groups successfully developed applications for rodents that supported folliculogenesis and the development of viable oocytes and births of healthy offspring [22–26]. These reports serve as proof of concept that artificial ovarian tissue can support folliculogenesis in small mammals. However, the extraordinary follicular growth in larger mammals makes it more challenging, and current ovarian scaffolds are insufficient [27]. Scaffolds derived from tissue-specific extracellular matrix (ECM) obtained by a concept known as decellularization received much attention in regenerative medicine and provided encouraging results for various organ/tissue reconstruction applications, including for uterine tissue [28]. Tissue-specific ECM-derived 3D-scaffolds have shown to influence mitogenesis, chemotaxis and to induce constructive host tissue remodelling and differentiation of endogenous stem cells [29, 30]. To our knowledge, there are only a few recent publications that explored decellularized tissues for ovarian bioengineering applications. For example, sliced bovine-, pig and human ovarian tissue have been decellularized and assessed for supporting structure for mixed primary ovarian cells [31–34]. The potential of this application was exemplified by restoring the hormonal function and initiating puberty in ovariectomized mice [31]. Furthermore, decellularized human skin was used in an attempt to improve graft vascularization and minimize the initial ischemic injury in two patients who underwent ovarian tissue transplantation [35]. Even if a healthy baby was born from this procedure, the true benefit of the scaffold was not verified. However, extracellular-rich scaffolds with Matrigel-alginate proved more favourable compared to fibrin-alginate scaffolds [36]. Collectively, these findings suggest that ECM scaffolds may be a good approach for ovarian tissue engineering. Various decellularization methods affect the recellularization ability, and consequently, the functionality of the constructed grafts. Sodium dodecyl sulfate (SDS) is commonly used as an effective decellularization reagent for many tissues and was mostly applied on ovarian tissue in earlier studies [31, 32, 34]. However, this chemical seems to compromise recellularization efficiency in several tissues [37, 38]. Moreover, using whole ovarian scaffolds instead of sliced structures may improve outcomes since it better mimics the natural 3D-structure and may allow a graft vascular anastomoses in future transplantation experiments that would reduce the initial ischemic injury. Therefore, the current study aimed to evaluate different decellularization protocols for whole mouse ovaries that later can be used as supporting structures for folliculogenesis in the rodent model, before moving towards studies in larger mammals.

## Materials and methods

### Donor animals for scaffold generation

A total of 83 female C57BL/6 N mice (Charles River, Germany) aged 10- to 20-weeks were used for the experiments. All animal work followed the local guidelines according to the approved ethical permit (114–2014; animal ethics committee at Gothenburg University, Sweden). Oophorectomy was conducted under isoflurane anaesthesia through an abdominal midline incision and the isolated ovaries were immediately put in Perfadex (Ex-vivo, Gothenburg, Sweden) and stored at − 20 °C before further processing.

### Ovarian scaffold generation by decellularization

Prior to the start of the decellularization procedures, all ovaries were independently weighed after the excess water had been removed by tapping each ovary on a dry filter paper. The initial decellularization attempts were conducted via low-pressure vascular perfusion through the ovarian artery (*n* = 35). However, no benefits were identified compared to the more direct and technically more suitable decellularization procedure which involved immersing the ovaries in the decellularization reagent and agitating at 100 rpm at room temp. Hence the later procedure was applied for all ovaries evaluated in this study.

We first assessed the efficiency of DNA removal by a 2% sodium deoxycholate solution (SDC) or a 0.5% SDS solution by measuring the total DNA quantification at various time-points after agitation. We also evaluated the decellularization efficiency using a combination of 1% Triton X-100 and 4% dimethyl sulfoxide (DMSO; 50% of the evaluated time for each solution) since this combination worked well for decellularizing rat uterus tissue [39, 40]. Promising decellularization protocols were then further optimized by adding enzymatic-, sterilization- and multiple washing steps.

The following three final protocols were selected for further evaluation; a) Protocol 1 (P1), 0.5% SDS for 10 h; b) Protocol 2 (P2), 2% SDC for 16 h; and c) Protocol 3 (P3), 0.5% SDS for 5 h followed by 2% SDC for 8 h (Table 1). For these three protocols, all ovaries were washed for 24 h in deionized water (dH_2_O) after their initial exposure to their respective detergents. They were then processed with an enzymatic step (DNase I; 40 units/ml; 30 min, 37 °C; Sigma-Aldrich, Stockholm, Sweden), followed by an additional 24 h wash with dH_2_O. The ovaries were then sterilized using 0.1% peracetic acid in 0.9% NaCl for 30 min, then washed six times for 5 minutes each with PBS, then for an additional 24 h in fresh PBS before being placed in new PBS + Gibco’s antibiotic-antimycotic (anti-anti; 1%; penicillin 10,000 U/mL, streptomycin 10,000 μg/mL and fungizone 25 μg/mL; Thermo Fischer Scientific, Gothenburg, Sweden) for long-term storage at − 20 °C.

**Table 1:** Summary of the final three decellularization protocols and subsequent sterilization procedure that were optimized in this study. Treatments below the dotted line were equal to all protocols

Details	Protocol 1	Protocol 2	Protocol 3
	SDS	SDC	SDS + SDC
Detergent 1	0.5% SDS (10 h)	2% SDC (16 h)	0.5% SDS (5 h)
Wash	dH_2_O (24 h)	dH_2_O (24 h)	dH_2_O (15 h)
Detergent 2	–	–	2% SDC (8 h)
Wash	–	–	dH_2_O (24 h)
Pre-treatment	PBS (1 h)		
Enzyme	40 IU DNase / ml of PBS (30 min; 37 °C)		
Wash	PBS (24 h)		
Sterilization	0.1% Peracetic acid in normal saline (30 min)		
Wash	Sterile PBS (6 × 5 min)		
Wash	Sterile PBS (24 h)		
Total time	85 h	91 h	103 h

*Abbreviations*: *SDS* sodium dodecyl sulfate, *SDC* sodium deoxychocolate, *dH*_*2*_*O* deionized water, *PBS* phosphate buffered saline, *h* hours, *min* minutes

### DNA quantification and electrophoresis

Homogenate from a decellularized whole ovary (*n* = 6 per protocol) and from a normal whole ovary (n = 6) was used for DNA quantification. DNA isolation was conducted with the DNeasy Blood and Tissue Kit (Qiagen, Stockholm, Sweden) according to the manufacturer’s instructions, and concentration was measured in triplicates on NanoDropOne (Thermo Fisher Scientific). The averages of total DNA (ng) per ovary and DNA per mass (ng DNA/mg ovary) were calculated. Electrophoresis was then performed to assess the size of the remaining DNA fragments in the decellularized ovaries for all groups and time points (*n* = 6), including normal ovary. For this procedure, a 1.5% agarose gel was run in Tris base acetic acid ethylenediaminetetraacetic acid (TAE) buffer with 1% GelRed (Bioticum Inc.,

San Francisco, CA, USA). Five μl from each extracted DNA sample and 1 μl of loading dye were loaded into the each well. As a reference trackIt™ 1 Kb DNA ladder (Thermo Fisher Scientific) was used. After running the gel (80 V; 70 min), the DNA was visualized and photographed using an Azure c600 (Azure Biosystems Inc., San Francisco, CA, USA).

### Total protein quantification

The protein contents were established from total homogenate from a whole decellularized ovary, and from a normal ovary for comparison. Total protein concentration was quantified with the Coomassie Protein Assay Kit (Thermo Fisher Scientific) according to the manufacturer’s instructions and read at 595 nm in a plate reader using a Bradford standard curve. Due to the small ovarian size and the low protein content in the decellularized- and normal ovaries, two specimens were pooled prior to analysis. Hence, from 6 ovaries per group we obtained an *n* = 3 for the statistical analysis.

### Histology and immunohistochemistry

For histology and immunohistochemistry-related analysis, four decellularized and normal ovaries from each group were used. Each sample was fixed in formaldehyde (1 h) and then dehydrated, embedded in paraffin and sectioned (5 μm sections). Sections were then dewaxed, rehydrated and processed with the following standard staining protocols; haematoxylin and eosin (H&E), Verhoeff van Gieson (for elastin), Masson’s trichrome (for collagen) and Alcian blue (for glycosaminoglycans; GAGs). Sections from each sample were also processed with 4′,6-diamidino-2-phenylindole (DAPI) to fluorescently label any potential DNA left in the tissue.

### Statistical analysis

The Shapiro-Wilk normality test was conducted and confirmed a skewed distribution of the data and therefore non-parametric tests were conducted. Thus, the shown values in graphs are medians with the respective interquartile range (IQR). The Kruskal-Wallis and Dunn’s multiple group comparison were conducted to evaluate significant difference levels (*p < 0.05, **p < 0.01, ***p < 0.001).

## Results

### Decellularization efficiency of various detergents on mouse ovaries

**Fig. 1:**
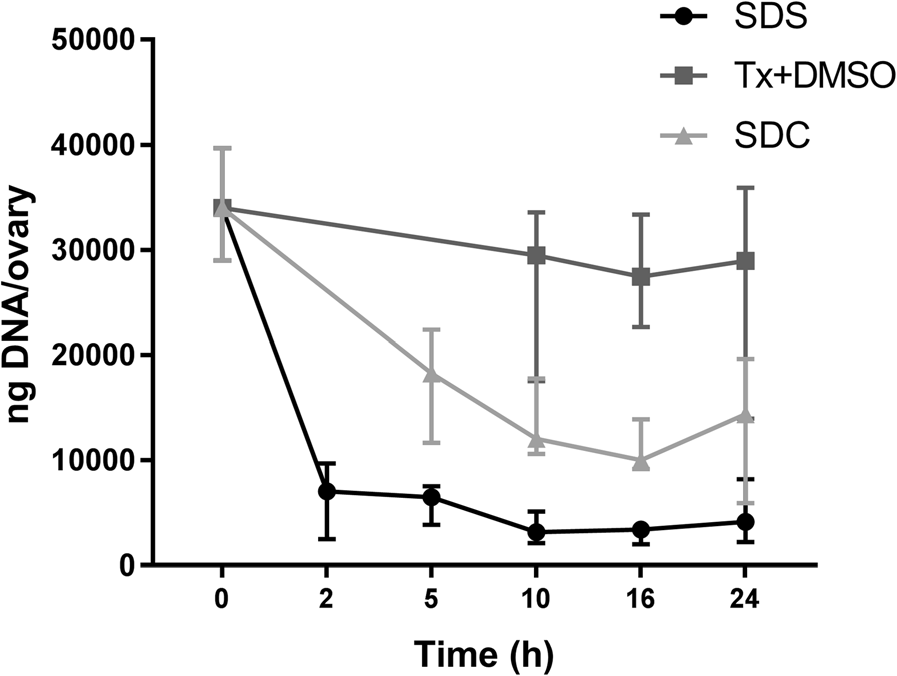
Efficiency of DNA removal from submerged and agitated normal mouse ovaries in different detergents during the pilot study first investigating the effectiveness of various decellularization chemicals during the first 24 h (*n* = 6 for all time points except for SDS_24h_ (*n* = 5), Tx + DMSO_10h/16h_ (*n* = 4) and SDC_24h_ (n = 5). The Shapiro-Wilk normality test suggested a non-parametric distribution. Thus, shown values are medians with the respective interquartile ranges. The Kruskal-Wallis and Dunn’s multiple group comparison were conducted to evaluate significant differences. Tx + DMSO, Triton X-100 and dimethyl sulfoxide

The initial evaluation of potential efficiency of decellularization detergents revealed that SDS and SDC were effective in removing DNA rapidly from mouse ovaries (Fig. 1). This process was much faster with SDS than to SDC or Triton X-100 + DMSO; a significant DNA reduction compared to the control was already seen at 2 h following SDS exposure (SDS_2h_; median value = 7028 ng/ovary vs. normal ovary, median value = 33,972 ng/ovary; *p* = 0.03; *n* = 6 per group). Due to the later decellularization effect seen in the SDC-group, we did not evaluate the amount of DNA after 2 h treatment. These results further confirmed that a combination of Triton X-100 and DMSO was not efficient for decellularization of mouse ovaries under these conditions. Same results were further confirmed by electrophoresis (not shown), which showed significant amounts of genomic size DNA remaining in the ovaries treated with Triton X-100 and DMSO. Much less DNA quantities (Fig. 1) with smaller fragments were observed in homogenate from SDC- or SDS- treated tissues. These initial results led us to evaluate the additional decellularization protocol using a combination of SDS and SDC (P3), and add an additional enzymatic treatment with DNase (employed on all ovaries in subsequent analyses) to further degrade the potentially immunogenic [41] lingering DNA fragments in the treated ovaries.

### DNA and protein quantification in decellularized ovaries

**Fig. 2:**
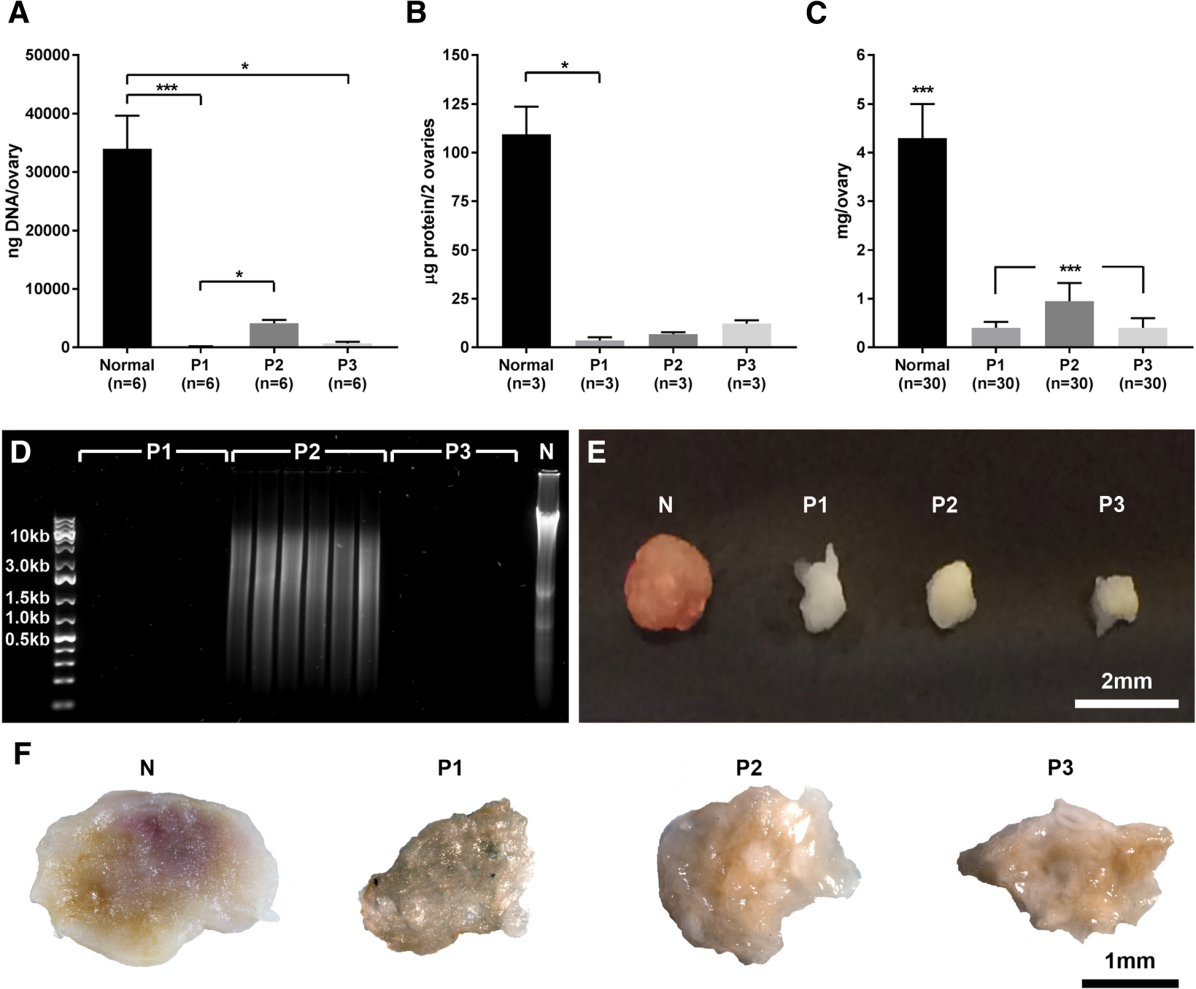
Successful decellularization was confirmed by a significant reduction in DNA levels for all protocols (**a**). Protein content was also reduced after decellularization (**b**), and the process affected the total weight of the ovaries (**c**). Gel electrophoresis confirmed low DNA concentrations, and lingering DNA fragments of various sizes were only detected in the SDC-treated (P2) ovaries (**d**). Furthermore, the protocol (P) 2 -treated ovaries exhibited less change compared with the P1- and P3-treated ovaries that were exposed to SDS during the decellularization, as indicated both by weight and macroscopic observations (**c**, and **e**-**f**) compared with normal (N) ovary. The Shapiro-Wilk normality test confirmed a non-parametric distribution. Thus, the shown values are medians with their respective interquartile ranges. The Kruskal-Wallis test and Dunn’s multiple group comparison were conducted to evaluate significant differences (*p < 0.05, **p < 0.01, ***p < 0.001). Note that values in B are based on two specimens per sample (a total of 6 ovaries per group were assessed in pooled pairs, resulted in *n* = 3 for statistical evaluation). Since all ovaries could be weighed, large samples groups were obtained for the weight assessment (C)

The DNA remnants and fragmentation sizes were further decreased by the addition of DNase in all protocols. The P1-treated ovaries exposed to the SDS treatment contained 145 ± 50 ng of DNA/ovary (median ± IQR; *n* = 6), which represented about 0.4% of its original DNA content (33,972 ± 5163 ng of DNA/ovary; median ± IQR; n = 6). For the SDC-treated ovaries in P2, the DNA content of the decellularized ovaries was 4146 ± 510 ng of DNA/ovary (median ± IQR; *n* = 6), which represented about 12% of its original DNA content. For the ovaries in P3 that were exposed to the combined treatment of SDS and SDC, the DNA content was 697 ± 442 ng of DNA/ovary (median ± IQR; *n* = 6) corresponding to about 2% of its original DNA content (Fig. 2A). The base pair size of lingering DNA was evaluated by running DNA-extracted homogenate from decellularized ovaries on a gel (n = 6 per group; Fig. 2D). This analysis confirmed undetectable amounts of DNA in the P1- and P3-treated ovaries. However, smeared bands appeared on the gel from all samples in the SDC-treated (P2) ovaries, indicating that lingering DNA in these ovarian scaffolds was of a variety of lengths, including large fragments.

A major reduction of the protein content could also be observed following all decellularization procedures (Fig. 2B). Note here, that the small ovarian size only allowed us to obtain detectable protein levels when two samples were pulled prior to analysis. Hence, from the 6 ovaries per group, this resulted in an *n* = 3 for the analysis and the subsequent statistical calculations. The SDS exposure caused by P1 significantly reduced the protein levels to a total of 3.6 ± 2.4 μg of protein/2 ovaries (median ± IQR; *n* = 3), corresponding to about 3% of the initial normal content (109.4 ± 11.5 μg of protein/2 ovaries; median ± IQR; n = 3). P2-treated ovaries contained about 6.9 ± 0.8 μg of protein/2 ovaries (median ± IQR; corresponding to about 6% of the original content; n = 3) and P3-treated ovaries had about 12.2 ± 4.6 μg of protein/2 ovaries (median ± IQR; about 11% of the original content; n = 3). Presumably due to low statistical power, the protein reduction measured in the P2- and P3-treated ovaries was not significantly lower than that of normal ovaries.

These significant effects caused by the decellularization methods were also confirmed by the measured weight loss of all the decellularized ovaries and by their obviously reduced size, as noticed by macroscopic observations (Fig. 2C and E). On average, and compared to the normal total ovarian weight prior to the decellularization (4.3 ± 1.4 mg/ovary; median ± IQR; *n* = 30), P1- and P3-treated ovaries both decreased by about 91% in weight. The P1-treated ovarian weight was 0.4 ± 0.4 mg/ovary and P3-treated total ovarian weight was 0.4 ± 0.3 mg/ovary; median ± IQR; n = 30 per group. The P2-treated ovaries were significantly heavier than both the P1- and P3-treated ovaries, with a weight of 0.95 ± 0.4 mg/ovary (median ± IQR; n = 30) which corresponded to about 22% of its original weight. The P2-treated ovaries also seemed more intact than the decellularized ovaries produced by other protocols (Fig. 2E-F).

### Histology and immunohistochemistry assessment on decellularized ovaries

**Fig. 3:**
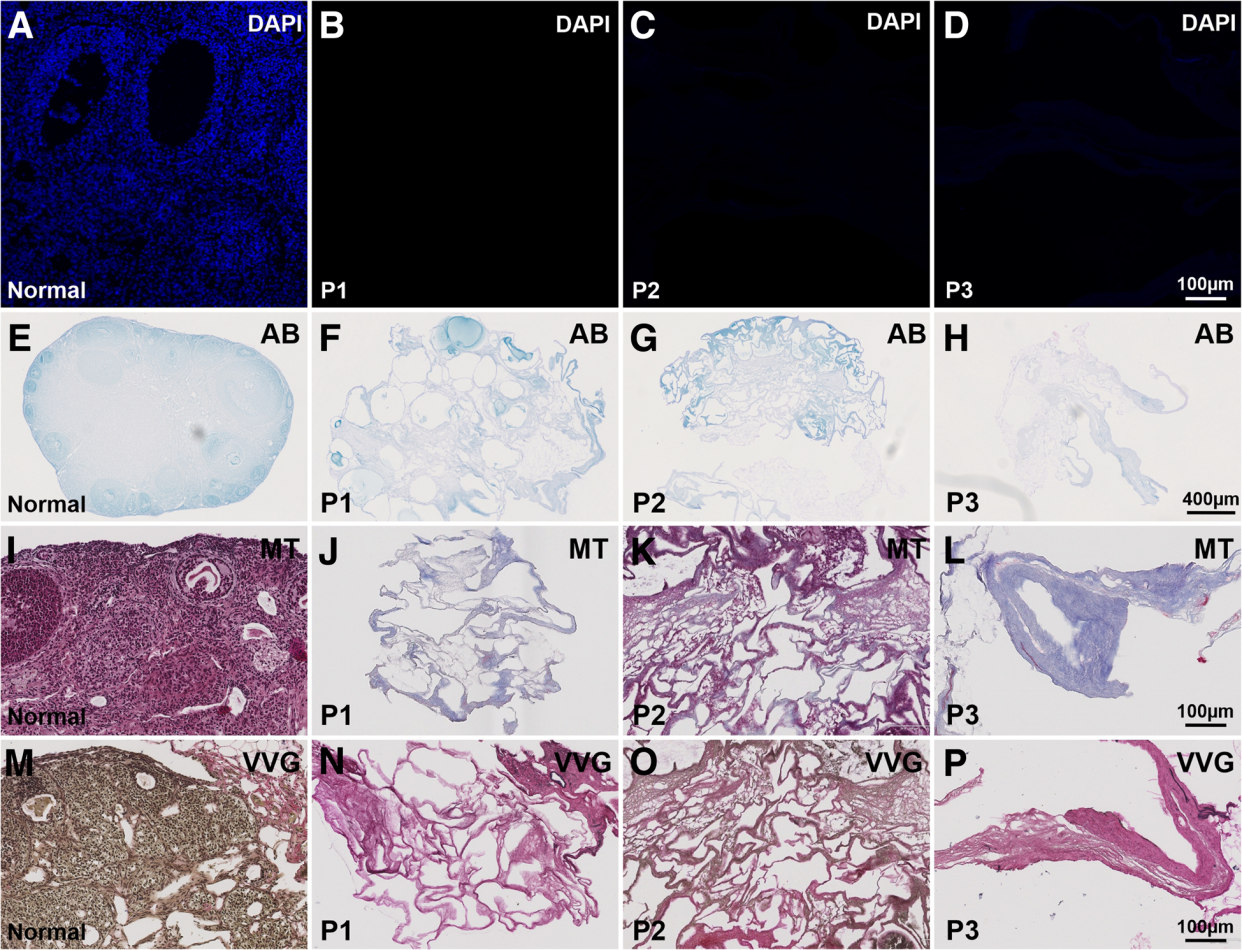
Fluorescently DAPI-stained tissue sections (blue; **a**-**d**) indicated a successful removal of DNA from the decellularized tissue and only a faint blue could be visualized in protocol (P) 3-generated scaffolds. Alcian blue (AB), which stains glycosaminoglycans showed that ovaries treated with the SDC-based P2 preserved the protein structures and maintained the original GAGs organization better compared to SDS-treated ovaries (P1 and P2; **f**-**h**). Masson’s trichrome (MT) staining showed preserved collagen structures for all ovarian scaffolds (blue; **j**-**l**). However, P2-treated ovaries stained a more distinct red-blue appearance that may indicate a higher presence of collagen and extra cellular keratin fibres. Verhoeff van Gieson staing (VVG; **m**-**p**) also suggested a better preserved ECM after the SDC-treatment (P2) compared to the other two protocols tested. No visual elastic fibres (stained black/brown; collagen stains red) appeared in the P1- or in P3-treated ovaries, whereas this was obvious for the stained ECM-structures from the ovarian scaffolds generated by P2 (N-P). DAPI, 4′,6-diamidino-2-phenylindole; GAGs, glycosaminoglycans; ECM, extracellular matrix; SDC, sodium deoxycholate

Morphologically, it was further evident that all the protocol treatments resulted in smaller ovaries with a less dense structure. Remaining cell nuclei were not visible in any of the protocols following multiple different staining methods (Fig. 3), including after staining with the sensitive fluorescent-based nuclear dye DAPI (Fig. 3A-D). The ovarian tissue seemed extensively damaged in the two protocols containing SDS (P1 and P3), especially in those ovaries exposed to both detergents (P3; Fig. 3H, L and P). However, the staining showed that the ECM was better preserved in the P1- and P2-produced scaffolds, where a porous structure could be observed in the cortex where follicles of various maturity may have been localized prior to the decellularization process. The ECM morphology was in general better organized following the SDC-treatment in P2 (Fig. 3G, K and O) compared with the other decellularization treatments. P3-derived ovarian scaffolds (Fig. 3H, L and P) had some ovary-like structures macroscopically, but their appearance had considerably changed morphologically.

Alcian blue, which is used to stain acidic polysaccharides like the ECM-rich GAGs, showed that ovaries treated with P1 and P2 preserved some of the original GAG structure better than other ovarian scaffolds produced by P3 (Fig. 3E-H). Masson’s trichrome staining showed preserved collagen for all the ovarian scaffolds, but again, with potentially higher amounts preserved in the P2-treated ovaries, as evidenced by the more distinct red-blue appearance that indicated a higher presence of collagen and extracellular keratin fibres (generally found in the ovarian surface epithelium; Fig. 3I-L). Verhoeff van Gieson staining also suggested a better organization of ECM after the SDC-treatment (P2) compared to the two protocols containing SDS (P1 and P3; Fig. 3N-P). This method stains elastic fibres and cell nuclei in black, while collagen fibres are stained red, and other tissue elements including cytoplasm are stained yellow (as can be seen in normal ovarian mouse tissue, Fig. 3M). No visible elastic fibres appeared stained in the P1- or P3-treated ovaries, however a prominent elastic- and collagen-rich ECM structure was visualized in SDC-treated ovaries (P2).

## Discussion

Ovarian bioengineering applications may be developed to aid in fertility treatment for patients that successfully have undergone cancer treatment but depleted their ovarian reserve as a negative consequence of treatment side-effects. Multiple research groups therefore studied ovarian bioengineering applications and successfully developed constructs based on hydrogels that supported folliculogenesis in small mammals that preserved fertility and lead to live pups following treatment [22–26]. However, these constructs have not yet succeeded to support large mammalian folliculogenesis, a process that requires a biomaterial with larger plasticity, where stromal cells and other cells of the normal ovarian compartment can aid in the support of the continuous growth and substantial expansion of the maturing follicles. It has been stipulated that biomaterials based on decellularized tissues may have these qualities [42] since it is based on the tissue-specific ECM that cells are able to integrate with and modify according to required needs for a growing follicle [43].

In line with this hypothesis, we here present novel data on the decellularization of whole mouse ovary using three different protocols. We assessed the decellularization reagents SDS, SDC and Triton-X100. These detergents are generally considered as strong, medium and weak decellularization reagents, respectively. Our results showed that SDS and SDC were effective and removed 99 and 88% of the original DNA, respectively. However, the week detergent (Triton-X100) combined with the ionic solution DMSO was not effective for the decellularization of mouse ovaries, at least not during the first 24 h. These negative results were unexpected since we earlier obtained good results using this chemical for the decellularization of the rat uterus where Triton-X100 + DMSO derived scaffolds proved effective for partial uterine repair in vivo [39, 40]. Thus, the findings of the present study importantly point out the difference of the structure of the internal genital organs and that protocols have to be systematically developed in order to optimize conditions, and that findings from one tissue cannot be extrapolated easily to another tissue.

One extremely well cited (more than 1500 times) research paper that reviewed results from 125 tissue engineering-related references concluded that lingering DNA in decellularized tissues should be less than 50 ng/mg of dry scaffold weight and not longer than 200 base pair to remain undetected from a negative host immune response [44]. Due to the impact of that review, these criteria seem to have become a general norm to achieve successful decellularization of tissues. Yet, there exist no clear scientific evidence that support these statements and the authors left out references from which these criteria were based on. Furthermore, studies have since emerged presenting positive in vivo results using decellularized tissues with larger amounts of donor DNA [40, 45]. It is therefore possible that the threshold is more forgiving than earlier postulated, or that this rather is a tissue specific phenomenon. Consequently, this should be established for every tissue engineering application and tissue type, in particular since remnants of the decellularization reagents in scaffolds also have negative effects [46]. Nevertheless, a third protocol was added to this study with the ambition to remove more DNA than the established SDC-protocol (P2). A combination of the two detergents SDS and SDC was therefore evaluated, but with the exposure time reduced by 50% for each detergent with the intention to remove more DNA than the SDC-based protocol while causing less damage to the ECM compared to the developed SDS-based protocol. As expected, this combination successfully removed more of the original DNA (about 98%), and led to undetectable DNA fragments of lingering donor DNA by electrophoresis. Thus, the ovarian scaffolds produced with this protocol, and by P1 pass the DNA size-criteria postulated in our earlier reference [44], while P2 left larger DNA fragments in the ovarian scaffolds than the proposed maximum size (< 200 base pairs).

However, the efficient DNA removal by the two SDS-containing protocols in the present study had a drawback, since the results of the present study clearly showed, both by the significant weight reduction and the observed morphological disorganization, that the SDC-based (P2) treatment preserved the ovarian ECM structure considerably better than the two other protocols using SDS. Additionally, a more distinct staining for GAGs (alcian blue stain), collagen (Masson trichrome stain) and elastic fibres (Verhoeff’s van gieson staing) were observed for the decellularized ovaries that had been exposed SDC instead of SDS. Furthermore, based on morphological observations, the combined treatment with both SDS and SDC (P3) were considerably negatively affected by the detergents during the decellularization process, and did not meet all the objectives we aimed for. Additional experiments that include the quantification of specific ECM molecules and assessing their supporting qualities for added cells and follicles after transplantation would provide meaningful information to determine if these constructs are suitable for future fertility applications.

In conclusion, the SDS-based P1 and the SDC-based P2 preserved the ECM structure after the decellularization procedures. The SDS-produced ovarian scaffolds (P1) contained minute amounts of donor DNA, a beneficial quality to evade a potentially detrimental immune response following engraftment. SDC-produced ovarian scaffolds (P2) contained more donor DNA but retained its extracellular composure better than ovaries treated with the SDS-based protocols. The SDC-based P2-produced ovarian scaffolds may therefore have improved recellularization capabilities, or other downstream bioengineering applications compared to SDS-developed scaffolds. However, we believe that this study presents two interesting scaffold candidates (P1 and P2) for future ovarian bioengineering experiments using the mouse model. These constructs may thus aid in evaluating the appropriateness and feasibility of using decellularized tissues as a potential support for maturing follicles that hopefully may lead to novel fertility preservation strategies to patients when ovarian cortex transplantation is not considered a safe treatment option.

## Data Availability

We acknowledge that protocols described in the manuscript, or any relevant raw data, will be freely available on request to any scientist wishing to use them for a non-commercial purpose.

## Abbreviations

3D: Three-dimensional
DAPI: 4′,6-diamidino-2-phenylindole
dH_2_O: Deionized water
DMSO: Dimethyl sulfoxide
ECM: Extracellular matrix
GAGs: Glycosaminoglycans
H&E: Hematoxylin and eosin
IQR: Interquartile range
P1: Protocol 1
P2: Protocol 2
P3: Protocol 3
POF: Premature ovarian failure
SDC: Sodium deoxycholate
SDS: Sodium dodecyl sulfate
TAE: Tris base acetic acid ethylene-diamine-tetraacetic acid
